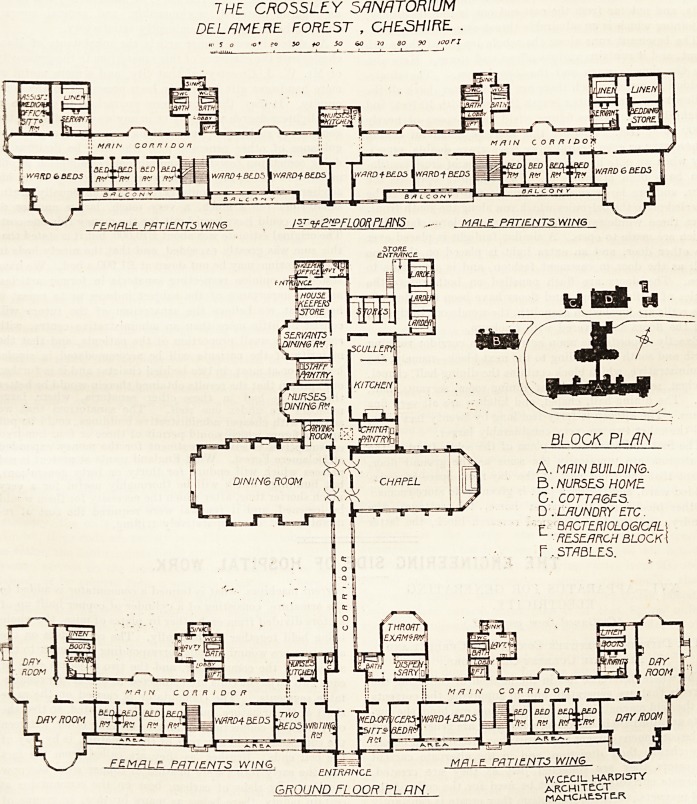# Crossley Sanatorium, Delamere Forest, near Frodsham and Mouldsworth Stations

**Published:** 1905-12-30

**Authors:** 


### HOSPITAL ADMINISTRATION.

### CONSTRUCTION AND ECONOMICS.

### CROSSLEY SANATORIUM, DELAMERE FOREST, NEAR FRODSHAM AND MOULDSWORTH STATIONS.

In a former number of The Hospital we gave a description of this splendid sanatorium, chiefly from the medical aspect, and we now publish the ground and first-floor plans, with a description thereof, written mainly from the architectural or rather constructional standpoint.

The building consists of seven blocks. The main, or the patients', block faces south-south-east. It is almost linear in plan, but is well broken up by bays, and there are large day-rooms at the ends with fine circular bays; and these, with the balconies and gabled roof of the centre, give an imposing and pleasing effect to the façade.

The main entrance is central. On the right hand on entering (the east end) are the medical officer's rooms, then a ward for four beds, staircase, four single-bedded rooms, and a very handsome double day-room. The west end has similar accommodation, save that the space occupied by the medical officer's quarters is here given up to a two-bedded room and a writing-room.

A wide, well-lighted corridor runs from one end to the other north of these rooms, and gives access to them all. At the north side of this corridor are several small blocks containing linen-rooms, boot-rooms, etc. The block containing the bath-rooms, lavatories, and closets is well arranged and is efficiently cut off from the corridor by cross-ventilated passages. Opposite the staircases are the lifts, and not far from the east-end one is the dispensary, adjoining which is an admirable throat-examination room.

The basement runs along the whole length of the south front, and it contains various offices and stores. Here also are the douche room and the *x*-ray room. The single-bedded rooms, of which there are twenty-four, have all the following measurements : length 15 feet, width 10 feet, and height 13 feet. These figures yield an air-space of nearly 2,000 cubic feet. Whether this extreme height of ceiling is necessary or even desirable we have grave doubts, especially when we note how carefully the means of cross-ventilation have been attended to. The windows open to the floor, and are large enough to enable the bedsteads to be wheeled on to the balconies which run along the south front. Over these windows or French doors are large fanlights, which are made to open. A similar fanlight is placed over the other door, and an extra light is placed on the same wall as the door in casement fashion, and is also made to open. The doors are flush panelled on both sides; the angles of walls, ceilings, and floors have been rounded off. The walls are finished in granolith, the woodwork in ripolin, and the floors are covered with linoleum.

Exactly opposite the main entrance is a corridor running north and south and leading to the next block—namely, the administrative, which block contains the dining-hall, chapel, kitchen, serving-rooms, nurses' dining-room, servants' hall, etc. The dining-hall, chapel, and kitchen are all very fine rooms. The kitchen is forty feet long by twenty feet wide, and the other two are very considerably larger.

The first and second-floor plans of the south front of the sanatorium are practically the same as the ground floor, except that the front part of the day-room space is a six-bedded ward, and the back part is given up to store-rooms.

Other blocks are the nurses' home, the cottages, the laundry, and the bacteriological research block, the latter containing post-mortem room, laboratory, and writing-room.

The nurses' home is placed west of the administrative block. It contains ample accommodation for a sufficient staff of nurses and servants, and very properly nothing has been overlooked which would be likely to ensure the convenience and comfort of the staff. In this block are the rooms provided for the matron and the housekeeper, and there are separate sitting-rooms and writing-rooms for the nurses and the domestics.

The sanatorium is lighted by electricity and is warmed by hot-water radiators and open fires. The building was designed by Mr. Cecil Hardisty, of Manchester, and as far as regards material, workmanship, and general appearance we believe it to be eminently satisfactory.

As stated in our former article the inhabitants of Manchester owe the existence of this sanatorium to the generosity of Mr. W. J. Crossley, of that city, and a more useful or more handsome gift has rarely been presented to any community. It may, therefore, seem ungracious to criticise in the slightest degree adversely or in any way to reflect on the goodness of such a gift, but we think we ought to say for the guidance of other generous men who may be desirous of doing something towards stamping out consumption in our midst, and not as any reflection on Mr. Crossley or on his advisers, that we are quite certain that an equally useful sanatorium for a much, a very much, larger number of patients, could have been built for the money this one cost. The original estimate was about £70,000, but it is stated that this sum was greatly exceeded, and that the ninety beds in the sanatorium may be put down at £1,000 a bed. We have stated our opinion respecting sanatoria in former articles, and the importance of the subject induces us to repeat it here, that we believe the sanatorium of the future will consist of little more than an administrative centre, with rooms for a small proportion of the patients, and that the majority of the patients will be accommodated in single-bedded, or at most, in two-bedded chalets; and it is further our opinion that the results obtained therein would be better than can be had in these other sanatoria, where large numbers are under one roof. The sanatorium that we advise, with cheaper administrative buildings, could be put up at a figure which would permit of three or four hundred consumptives receiving treatment for the money expended at Delamere Forest. What England wants at present is not palaces which will endure for thirty or forty generations, but buildings which will be thoroughly useful for a very much shorter time, after which the necessity for them would be lessened, and if renewal were required the cost of renewal would be comparatively trifling.

## Figures and Tables

**Figure f1:**